# Progressive Muscle Cell Delivery as a Solution for Volumetric Muscle Defect Repair

**DOI:** 10.1038/srep38754

**Published:** 2016-12-07

**Authors:** Ji Hyun Kim, In Kap Ko, Anthony Atala, James J. Yoo

**Affiliations:** 1 Wake Forest Institute for Regenerative Medicine, Wake Forest School of Medicine, Winston-Salem, NC 27157, USA

## Abstract

Reconstructing functional volumetric tissue *in vivo* following implantation remains a critical challenge facing cell-based approaches. Several pre-vascularization approaches have been developed to increase cell viability following implantation. Structural and functional restoration was achieved in a preclinical rodent tissue defect; however, the approach used in this model fails to repair larger (>mm) defects as observed in a clinical setting. We propose an effective cell delivery system utilizing appropriate vascularization at the site of cell implantation that results in volumetric and functional tissue reconstruction. Our method of multiple cell injections in a progressive manner yielded improved cell survival and formed volumetric muscle tissues in an ectopic muscle site. In addition, this strategy supported the reconstruction of functional skeletal muscle tissue in a rodent volumetric muscle loss injury model. Results from our study suggest that our method may be used to repair volumetric tissue defects by overcoming diffusion limitations and facilitating adequate vascularization.

Cell-based therapies in tissue engineering (TE) and regenerative medicine (RM) provide promise to restore normal functions of damaged and injured tissues and organs^1^. Such strategies include cell transplantation and implantation of engineered tissue constructs, where efficient cell survival following implantation is a critical factor to the success. Cell-based strategies have been used successfully in preclinical and clinical trials to treat defects in avascular tissues, such as cartilage and cornea, which do not necessitate blood supply to maintain cellular viability and function under hypoxic conditions^2^,^3^,^4^. Small injuries in the vascularized tissues that correspond to a few microns can be repaired using cell-based approaches because the implanted cells will remain viable due to direct transport of oxygen and nutrients within 200 μm^5^,^6^,^7^,^8^,^9^ from host vasculatures as well as diffusion from adjacent host blood vessels. Skin regeneration has been achieved using cell-based therapy;^10^,^11^ however, efficient treatment of defects larger than millimeter or centimeter scale in vascularized tissues and organs such as heart, liver, and skeletal muscle remains challenging. In most cases, repair of larger tissue defects requires implantation of large, volumetric engineered tissue constructs or implantation of high-dose cells^12^,^13^,^14^ to restore normal functions. Under such conditions, oxygen transport to all of the implanted cells is difficult. In particular, cells located in the center of thick tissues (a few millimeter scales) with low oxygen concentration will become necrotic leading to failure of tissue grafts. To improve the cellular viability within large-sized defects, efficient nutrient and oxygen supply are necessary;^1^,^15^,^16^ therefore, strategies need to be developed for volumetric tissue repair to improve vascularization, which will have a positive impact on cell survival.

To date, several strategies have been developed to accelerate vascularization of engineered tissues. The conventional method used in early studies promoted vascularization for survival of the implanted cells through stimulation of *in vivo* microenvironments at the time of implantation. To stimulate vascular environments, pro-angiogenic factors such as vascular endothelial growth factors and fibroblast growth factors were incorporated with engineered tissue constructs, followed by the implantation^17^. In other cases, exogenous endothelial stem or progenitor cells were co-seeded with tissue-specific cells before implantation^18^,^19^. Although incorporation of such vascularization cues resulted in improved vascularization *in vivo*, formation of new blood vessels within the implant site was too slow to support the majority of implanted cells^7^,^16^,^19^,^20^. Pre-fabrication of vascular networks within engineered tissue constructs during *in vitro* cell culture of the seeded scaffolds provides an alternative strategy for the repair of a volumetric muscle defect. Morphological characterization has revealed that *in vitro* pre-vascularized tissues contained well-organized vascular structures and could accelerate vascularization time by providing adequate blood supply to the seeded cells. Unfortunately, host-implant anastomosis of *in vitro* pre-vascularized tissues usually occurs within several days after implantation;^21^,^22^,^23^ thus, integration of reconstructed tissue with the host was inefficient. An *in vivo* pre-vascularization strategy has been developed to fabricate large-sized, vascularized implantable constructs. By implanting the cell-seeded scaffold into the highly vascularized site, vascular tissues could be obtained *in vivo* and transferred to the target site^24^,^25^,^26^,^27^. In another study, the polysurgery approach was proposed to produce thick, viable myocardial tissues at an ectopic site^28^. This work shows that repeated cell-sheet transplantation at time intervals of 1–2 days can generate vascularized cardiomyocyte sheets *in vivo*. While those strategies is a promising approach in terms of addressing volumetric tissue defects, several issues such as delayed perfusion, numerous surgical interventions, and inefficient cell grafting within the vascularized explanted tissue must be addressed before clinical use^15^. Therefore, none of the conventional vascularization strategies is appropriate for volumetric tissue repair.

Towards this end, we proposed a novel and simple cell delivery method that enables reconstruction of viable, large tissues *in vivo* for restoration of volumetric tissue injury through an efficient vascularization strategy. As described above, conventional cell-based approaches for volumetric tissue repair are limited due to inefficient blood supply for implanted cells. Therefore, we hypothesized that multiple injections of a high dose of cells in a progressive manner would maintain cellular viability through the vascularization process when compared to single injection of the same number of cells for implantation. We utilized the normal vascularization process that occurs during the natural regeneration process (Fig. 1). To show the feasibility of restoring functional volumetric tissues in the defect site, multiple, progressive delivery of cells was performed using ectopic cell transplantation in a subcutaneous site. Appropriate cell delivery parameters such as cell density, cell injection volume, and time interval between injections were tested. The efficiency of volumetric tissue formation was compared with single injection of the same number of cells that were used for multiple injections. Furthermore, this cell delivery technique using C2C12 cells and human muscle progenitor cells (hMPCs) was applied to a rodent volumetric muscle loss (VML) model; moreover, histological and functional recovery was evaluated to determine the possibility for applications to treat critical-size muscle defects.

## Results

### Ectopic muscle construction by multiple and progressive cell injection

To investigate the feasibility of restoring volumetric muscle tissues by multiple cell injections in a progressive manner, C2C12 cells were subcutaneously injected in athymic mice, and the volume of the newly formed tissues was measured at pre-determined injection points for comparison. Multiple-cell injections with one week interval between each injection resulted in an increased volume of the implants (see Supplementary Fig. S2a). Quantitatively, increased number of cell injections (up to 8 injections) correlated with an increased implant volume (Fig. 2a). Particularly, 6–8 cell injections demonstrated a statically significant difference (ANOVA and *post hoc* Tukey Test) when compared with 2–4 cell injections (*P < 0.0001 and ^†^*P* < 0.005, respectively). The volumes of implants in all multiple, progressive cell injected groups showed a significant increase compared to the progressive gel only-injected groups (ANOVA, ^‡^*P* < 0.001). In hematoxylin and eosin (H&E) and masson’s trichrome (MT) staining images (see Supplementary Fig. S2b), increase in the volume of reconstructed tissue formation was notable between 2 and 4 cell injections, but no significant size difference was observed beyond 4 cell injections. In each group, the MT images demonstrated that the newly formed tissue structures were skeletal muscle fibers as confirmed by red staining within the implant.

The efficiency of progressive cell delivery in the reconstruction of volumetric tissue was determined by comparing to the single injection of cells. In this comparison, total volume (1.2 ml) and cell number was set to be equal between multiple (4 × 0.3 ml per injection) and single injection (1.2 ml for one injection); furthermore, 4 progressive cell injections were selected as more than 4 injections would yield an unimplantable volume for single injection to the animal. The volume of the reconstructed tissue of the progressive cell injection group showed approximately 4-fold increase as compared to that of the single injection of cells with a statistical difference (Student’s *t*-test, *n* = 4, *P < 0.0001) (Fig. 2b). Interestingly, the results of green fluorescent protein (GFP) immunostaining revealed that the progressively-injected C2C12 cells contributed to the formation of muscle fibers *in vivo* as confirmed by GFP^+^ muscle fiber-like structures within the reconstructed tissue (Fig. 2c), while a few GFP^+^ C2C12 cells scattered in the implant were found in the single injection. This result demonstrates that multiple, progressive cell injections are more effective than single injection for the formation of a volumetric muscle-like structure at a muscle ectopic site.

### Efficient muscle cell survival, muscle tissue formation, and vascularization by progressively injected cells

To examine the muscle tissue formation and maturation of progressively injected C2C12 cells, myosin heavy chain (MHC) immunostaining was performed on the reconstructed tissue. Throughout the reconstructed tissue, viable MHC^+^ muscle fiber-like structures and cells were clearly visualized as indicated by the dotted lines (Fig. 3b, first row). In the area of the early 1^st^ injection (inner site of the implant), a number of MHC^+^ multi-nucleated muscle fibers were observed, while a few MHC^+^ cells with pre-matured structures were found in the area of the last 4^th^ injection (outer site). The observable difference in muscle maturation demonstrates that multiple cell injections performed in a progressive manner allows muscle cell survival, muscle formation, and maturation of the injected cells in an ectopic site.

Vascularization of the injected C2C12 cells in a timely manner is a critical factor to re-create volumetric muscle tissue by providing an adequate blood supply to the grafted cells. We hypothesized that each injection of cells in a progressive manner would promote efficient vascularization with the host. To test the hypothesis, double staining of GFP and von Willebrand factor (vWF) was performed to determine whether the injected GFP^+^ C2C12 cells are localized with vWF^+^ blood vessels for improved cell survival. The double fluorescent imaging showed that GFP^+^ C2C12 cells are present throughout the injected area and are localized within 100–200 μm of vWF^+^ blood vessels (Fig. 3b, second row). This finding suggests that progressively injected cells remained viable along with newly-formed vasculatures in the injected area up to 4 weeks after injection.

### Vascularization and neuronal ingrowth by progressive cell injections

To compare vascularization outcomes between the progressive and single cell injections, vWF/α-smooth muscle actin (α-SMA) immunostaining was performed in each group (Fig. 3a,b). Quantitatively, new blood vessel formation and vascular maturation were assessed by counting the number of vWF^+^ vessels (per field), and calculating the area of vWF^+^ vessels (μm^2^ per field), maturation index (%) and percentage of different vessel sizes (Fig. 3c, *n* = 4, 9–12 fields per each sample). The number and area of vWF^+^ vessels (/field) in the progressive injection samples significantly increased, as compared to that of the single cell injection group (Fig. 3c-i,c-ii, Student’s *t*-test, *P < 0.05). However, the level of vascular maturation showed no significant difference between the two groups (Fig. 3c-iii, Student’s *t*-test, *P* > 0.05). The majority of blood vessels observed in the single cell injection group was less than 200 μm in diameter, whereas a higher percentage of larger blood vessels greater than 500 μm in diameter was present in the progressive cell injection group (Fig. 3c-iv, Student’s *t*-test, *P < 0.05). These results indicate that multiple, progressive cell injection is an effective cell delivery method to achieve vascularized volumetric muscle structure.

To examine whether vascularization and maturation during progressive cell injections occur in a normal and physiological manner, blood vessels formation of progressively injected areas (the 1^st^, 2^nd^–3^rd^, and 4^th^ injected areas) was quantified by using vWF/α-SMA immunostaining images (Fig. 3d, *n* = 4, 3–5 fields per each area in each sample). Large and matured vessels (vWF^+^ α-SMA^+^) were observed in both areas surrounding the 1^st^ injection site and the 2^nd^–3^rd^ injection (Fig. 3b, third row). More immature vessels (vWF^+^ α-SMA^−^) are visualized in the area of the 4^th^ injection (last injection) than any of the other sites. The site of the 4^th^ injection showed the highest number of vWF^+^ blood vessels and the lowest area of vWF^+^ vessels with a significant difference (Fig. 3d-I,d-ii, ANOVA, *P < 0.05 with the 1^st^ injection, ^†^*P* < 0.05 with the 2^nd^ injection, Tukey test), which demonstrated more newly formed small-sized capillaries at the injection site when compared to that of the earlier injected sites. In terms of the degree of blood vessel maturation, the 1^st^ injection site (inner site) showed highest maturation index compared with the other three sites (Fig. 3d-iii, ANOVA, *P < 0.05 with the 1^st^ injection, ^†^*P* < 0.05 with the 2^nd^ injection, Tukey test). These quantitative results correlate with the percentage of blood vessels as a function of blood vessels size (Fig. 3d-iv). This finding is consistent with normal angiogenesis that can be found in another study^29^. In contrast, inner area of implants constructed by single cell injection showed the lowest area of vWF^+^ vessels, and there were no significant differences in number, maturation and percentage of blood vessels among inner, middle, outer areas (see Supplementary Fig. S3). In addition, neuronal ingrowth, which is a critical cellular event in functional muscle regeneration, appeared to occur in a normal condition. The progressive cell injection facilitated recruitment of Neurofilament (NF)^+^ peripheral nerves at each injection site (Fig. 3b, lowest row). These results showed that multiple cell injections in a progressive manner maintained viability of the delivered cells within the muscle ectopic site while single injection did not; furthermore, the vascularization and neuronal ingrowth were observed that facilitated the volumetric skeletal muscle construct *in vivo*.

### Improved structural and functional recovery of critical defect in TA muscle by progressive cell injection

Encouraged by promising outcomes in the ectopic implantation study, we applied the progressive cell delivery method to restore muscle function of the injured skeletal muscle. To test the possibility, a volumetric muscle defect in a critical-size was utilized to test the efficiency of the progressive cell delivery method. As a volumetric muscle defect model, 30% of original tibialis anterior (TA) muscle mass was excised and this level of muscle loss is incapable of fully restoring the TA muscle for several months without any treatment. Therapeutic efficacy of progressive cell injections was evaluated by anatomical and functional analysis (*n* = 4 per each group). Grossly, the TA muscle of progressive injections was harvested at 4 weeks after first injection and showed better TA anatomy than no treatment (defect only), gel only, or single injection group (see Supplementary Fig. S4). As observed in the gross images, single injection of C2C12 recovered the defected TA muscle by a 1.3-fold increase in TA muscle mass compared with no treatment and gel only injection group (Fig. 4a). The ratio of the treated- to contralateral TA muscle mass of the progressive C2C12 injection group showed a 1.3-fold increase compared to that of the single C2C12 injection group with a significant difference (ANOVA and Tukey test, ^§^*P* < 0.017) (Fig. 4a). Interestingly, the increased muscle mass correlates with improved muscle function. Tetanic muscle force of the progressive C2C12 muscle injection group showed a 2-fold increase, as compared to that of the single C2C12 injection group (ANOVA and Tukey test, ^§^*P* < 0.016). Muscle function improvement demonstrated by the progressive C2C12 injections was approximately 42% of normal TA muscle. The multiple progressive injections of hMPCs also showed a similar pattern, in terms of muscle function improvement. When compared with the single hMPC injection, the progressive hMPC injections facilitated a 1.5- and 2.6-fold increase in muscle mass and function, respectively (Student’s *t*-test, *P < 0.05) (Fig. 4c). Progressively injected hMPCs in the TA muscle showed 39% restoration of the muscle function, as compared with normal TA muscle. Improved functional outcome by both cell types was evidenced by histological analysis. Based on the H&E images, TA muscle thicknesses of progressive C2C12 and hMPC injection groups were 1.6- and 1.2-fold higher than that of single injections with a statistical difference (ANOVA and Tukey test, ^§^*P* < 0.001 and Student’s *t*-test, *P < 0.003) (Fig. 4b,d), respectively.

To evaluate the level of fibrosis in muscle tissue, collagen I/MHC immunostaining was performed. The percentage of collagen I^+^ area of inner TA and outer TA was quantified (*n* = 4, 3 random fields per each area in each sample) (see Supplementary Fig. S5). In all groups, severe fibrosis was observed in the outer area of injection sites, when compared with the inner TA. However, the progressive injection group, regardless of the cell types, resulted in reduced levels of fibrosis, as compared with the single cell injection group in both the inner TA and outer TA (ANOVA and Tukey test, *P* < 0.05). Particularly, the degree of fibrosis of the inner area in the progressive C2C12 injection group was comparable to normal TA muscle with no statistical difference (ANOVA and Tukey test, *P > 0.997). Overall, multiple cell injections in a progressive manner is an effective cell delivery method in term of anatomical and functional improvement of volumetric muscle defect, when compared with no treatment, cell delivery vehicle only and single delivery of same cell number.

### Muscle fiber formation of progressively injected cells and vascularization and neuronal ingrowth

To examine whether each progressive cell injection can efficiently deliver the infused cells to occupy the defect space within the TA muscle defect site, the GFP^+^ C2C12 cells were fluorescently labeled before implantation, and the localized cells were tracked by a double fluorescent imaging based on GFP expression and fluorescent labeling. Progressively-injected C2C12 cells showed distinguishable localization within the TA defect site (Fig. 5a) where the cells were fluorescently labeled only for 1^st^ and 4^th^ injection. In the double GFP^+^ fluorescent labeling imaging, fluorescently labeled C2C12 cells at 1^st^ and 4^th^ injection site were clearly visualized as red fluorescence (yellow arrows along with dotted lines in whole TA) while cells injected during the 2–3^rd^ injection were stained with GFP without red fluorescent labeling. Single injection of C2C12 cells, which represents the same cell number as the total cell number delivered by progressive injections, also showed a few fluorescent signals in the outer part of the TA muscle. In magnified images, the number of injected cells with fluorescently labeled GFP^+^ MHC^+^ C2C12 cells (white arrows) is higher with progressive injections than that by single injection (Inner TA). In the inner part of the TA muscle (Fig. 5a), the majority of infused C2C12 cells by progressive injection formed muscle fibers (MHC^+^ staining) and newly formed muscle fibers (chimeric) (MHC^+^ GFP^+^ labeling, as indicated by white arrows) organized with the host muscle tissue. The outer site of the TA muscle showed different cell localization compared with the inner site. In the progressive group, a number of injected C2C12 cells with fluorescent labeling are found to contribute to the muscle formation with host muscle tissues (white arrows). More interestingly, the number of GFP^+^ MHC^+^ fluorescent-labeling positive cells is higher in the progressive injection group than in the single injection group. In addition, a few fluorescently labeled-GFP^+^ C2C12 cells at the surface site of the TA muscle are not involved in the muscle formation as indicated by white arrowheads. hMPC injection also showed a similar pattern of cell distribution with C2C12 cells (Fig. 5b). Progressive injection of hMPCs resulted in efficient distribution at the entire site of the TA muscle, as confirmed by double staining of MHC and human leukocyte antigen (HLA) staining (Fig. 5b, lower row). More HLA^+^ MHC^+^ chimeric muscle fibers are clearly found in both inner and outer TA site (white arrows) by progressive injection but not with single injection. Interestingly, some of chimeric muscle fibers (white arrows) in the progressive group in the outer area of the TA muscle tissue are notable in terms of clear involvement of the injected hMPCs (white arrows in outer TA).

Differentiation of injected C2C12 cells and hMPCs into myofibers or myotubes was quantified by calculating the percentage of fluorescent-labeled-Myogenin^+^ C2C12 cells and Myogenin^+^ human nuclear antigen (HNA)^+^ cells in the C2C12-injected group and hMPCs-injected TA muscles, respectively (*n* = 3–4, 3–5 fields per sample). In both C2C12-injected group and hMPCs-injected TA muscle, the percentage of fluorescently labeled Myogenin^+^ cells and Myogenin^+^ HNA^+^ cells was higher with progressive injections than that by single injection with a statistical difference (Student’s *t*-test, *P* < 0.05) (42.74% ± 10.63% and 19.18% ± 6.04% in progressive C2C12 and hMPCs injection, respectively) (see Supplementary Figs S6 and S7). Notably, it was clearly seen that fluorescently labeled C2C12 cells were fused to form muscle fibers with host muscle tissues, some of which was Myogenin^+^ cells. Proliferation of injected cells in the TA muscles was evaluated by fluorescent staining images of proliferating cell nuclear antigen (PCNA). The percentage of proliferating C2C12 cells (fluorescent labeled PCNA^+^) and hMPCs (PCNA^+^ HNA^+^ cells) in the progressive injection group was 33.31% ± 15.56% and 31.99% ± 15.84%, respectively, which is higher than in the single injection group (*n* = 3–4, 3–5 fields per each sample, Student’s *t*-test, *P* < 0.05) (see Supplementary Figs S6 and S7).

To examine the vascularization and neuronal ingrowth by the injected hMPCs, vWF/α-SMA and NF/AChR/MHC immunostaining were performed, and double- and triple-positive staining was visualized, respectively. Quantitative results of vascularization indicated that only progressive hMPCs injections showed increased vascularization, in terms of number and area of vWF^+^ vessels (/field) (*n* = 3–4, 6–10 fields per sample, ANOVA and Tukey test, *P* < 0.05) (Fig. 6b,c), while the degree of vascularization was not statistically different among the no treatment, gel only and single hMPCs injection groups (ANOVA and Tukey test, *P* > 0.05). Meanwhile, the degree of vascularization between inner region and outer region in each group was not statistically different in terms of the number and area of vWF^+^ vessels (/field), maturation index (%) and percentage of different vessel sizes (data not shown, Student’s *t*-test, *P* > 0.05). Neuronal ingrowth of the TA muscle was confirmed as NF^+^/AChR^+^/MHC^+^ staining in both progressive and single injection, and the level of neuronal ingrowth was not significantly different between the injection methods (see Supplementary Fig. S8).

## Discussion

With increasing interests in translation of cell-based therapies from pre-clinical to clinical applications, appropriate treatment of large volumetric tissue defects relies on efficient cell survival following implantation^15^,^30^. While several cell-based approaches using TE and RM techniques have facilitated successful outcomes in terms of recovery of avascular tissue function through an effective tissue regeneration conditions^2^,^3^,^4^, reconstruction of volumetric and highly-vascularized tissues or organs on a large scale (>mm to cm) *in vivo* remains challenging. Decreased cell survival of the implanted cells due to an insufficient blood supply to the implanted cells has limited the efficient integration of such large constructs with the host vascularization^1^,^15^,^16^. To address this issue, several pre-vascularization approaches have been developed and many have demonstrated successful reconstruction of vascularized tissues in *in vitro* and *in vivo*^15^,^31^. The time-consuming vascularization process, however, must be overcome to allow for the volumetric repair^15^,^30^. Currently, efficient method is available to treat the volumetric tissue defect; therefore, we developed an effective cell delivery method to construct a large size muscle tissue through an efficient vascularization process *in vivo.* Our results from histological and immunohistochemical analysis demonstrate that muscle cell delivery by multiple cell injection in a progressive manner facilitated large scale muscle (>mm) tissue formation in an ectopic mouse model, and the reconstructed tissue is well-integrated with the host vascular system and neuronal ingrowth. When this technique was applied to a skeletal muscle injury model with a critical volumetric defect, the progressive cell injection demonstrated enhanced muscle tissue reconstruction and improved functional recovery when compared with single injection. From these results, we suggest that our cell delivery system in a multiple progressive manner is a promising and effective method to reconstruct volumetric muscle and thereby improve muscle function *in vivo*.

**Figure 1:**
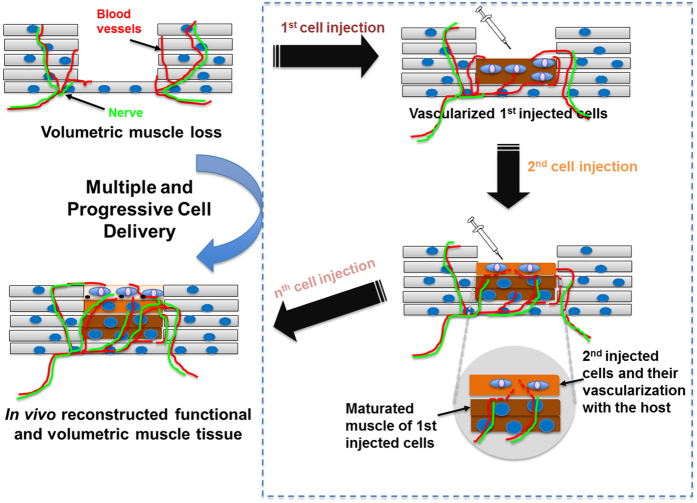
Schematic diagram of multiple cell injections in a progressive manner for functional and volumetric tissue reconstruction.

The idea of ‘multiple and progressive cell delivery’ for volumetric tissue reconstruction arose from the phenomena frequently observed in cell-based approaches;^32^ which includes decreased cell survival of transplanted cells or implantation of engineered tissue construct due to the insufficient delivery of oxygen and nutrients as a result of the time-consuming process of vascularization. Large defect sizes (e.g. mm-cm in size) are particularly affected by decreased cell viability when in the vascularized tissues needs to be repaired. In this case, a high number of cells are usually required for treatment, and the requirement for oxygen and nutrients increases with the increased cell number^1^. To address this critical issue, we hypothesized that a multiple number of cell injections performed in a progressive manner would effectively enhance volumetric tissue *in vivo* (Fig. 1). This delivery method is primarily designed to improve the viability of the delivered cells at each injection to allow for efficient vascularization surrounding the cells at the delivery site. The first injection of cells at an appropriate cell number and injection volume will obtain sufficient oxygen from the host vessels at the injection site. Following the first injection, the host vasculature will surround the site of the injection within a few days and provide vascular beds for subsequent cell injections. The period of vascularization following each subsequent injection results in the formation of a suitable angiogenic environment for the injected cells. Repeating the cell injection process allows a larger and thicker tissue with structural and functional properties to be reconstructed *in vivo* (Fig. 1).

To test the hypothesis, we utilized an ectopic implantation model through subcutaneous injection in athymic mice. The subcutaneous injection model was chosen for several reasons including a highly vascularized, non-myogenic tissue that was easily accessible for cell injection. It was particularly important to evaluate muscle regeneration within a non-myogenic environment to evaluate whether the volumetric muscle formation *in viv*o occurred as a result of the injected muscle cells following the progressive cell injection strategy and without any contribution of host muscle tissue. Using this model, we first attempted to optimize cell density for injection. Subcutaneous injection of three different cell concentrations showed dramatically different results in terms of cell survival, where highest cell density (30 × 10^6^ cells per ml) indicated higher necrosis with an exponential increase (see Supplementary Fig. S1), thus, indicating that a higher cell density within the implanted area exceeded the amount of oxygen and nutrients that could be supplied by the host. It is plausible that high cell concentration increased oxygen consumption by cells, while diffusion of oxygen decreased in denser tissues. Therefore, cell concentration of 10 × 10^6^ cells per ml was selected for this study to prevent necrosis and improve tissue formation.

In addition to the selection of an appropriate cell density for the cell injection, the time interval between each cell injection was an important parameter to be considered. As a proof-of-concept study, we chose an interval of one week between each of the multiple cell injections based on the time for normal vascularization to occur in the body^29^. Generally, the new vascularization surrounding the cell injection site will occur within one week and new blood vessel formation under maturation will occur at 2–3 weeks. We attempted to perform four series of cell injections in a progressive manner in the ectopic implantation study (Fig. 3); therefore, we expected that the 1^st^–3^rd^ injection, which occurred at least two weeks before harvesting the reconstruct tissue formation, would form the appropriate vascular maturation while the 4^th^ (final) injection site would show newly formed capillaries. The results of immunostaining and quantification analysis confirm that one week between each injection established an appropriate time interval to produce efficient vascularization along each cell injection (Fig. 3b,d). Our results are consistent with the outcomes from another study that established a vascular chamber *in vivo* using an arterio-venous loop (AV loop) and optimized the timing of cell implantation to determine efficient cell survival^33^. Their results showed that angiogenic activity peaked between 7–10 days after insertion of the AV loop and suggested that as further vascularization led to an increased survival among the implanted cells. This study revealed that delayed cell implantation (at day 7) into a site with well-established vessels could improve cell survival^33^.

**Figure 2:**
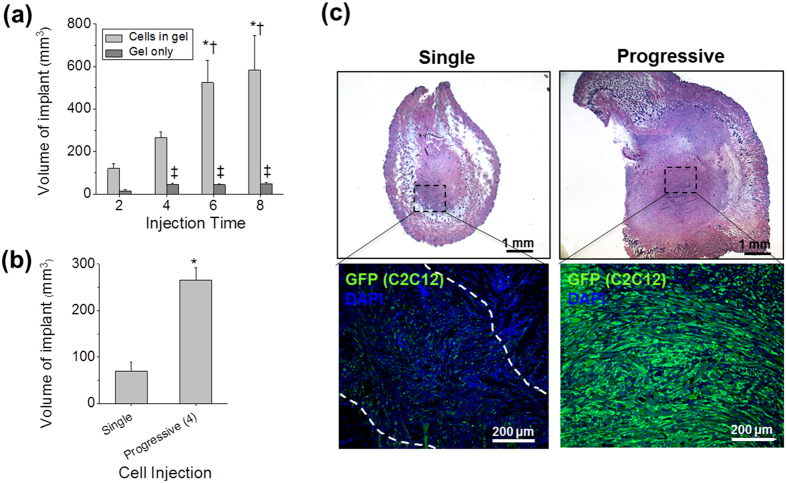
Ectopically implanted cells survival and their volumetric tissue construction *in vivo*. (**a**) Multiple cells in gel or gel only injections in a progressive manner were performed with athymic mice and the volume of implant was measured by injection time (2, 4, 6, and 8 injections). Each injection of GFP^+^ -C2C12 was performed once in a week with the equal volume of cells in gel or gel only. ANOVA, Tukey test (*n* = 3–4). *P < 0.0001 with 2 times of progressive cell injection, ^†^*P* < 0.003 with 4 times of progressive cell injection, ^‡^*P* < 0.001 with 2 times of gel only injection. (**b**,**c**) The efficacy of progressive cell delivery was compared with single cell delivery for implanted cells survival and volumetric tissue construction. Total injected volume of 4-progressive cells in gel injection was same as that of single injection. Volume of implant (mm^2^) of single and 4-progressive injection was compared in (**b**). Student’s *t*-test (*n* = 4). *P < 0.0001 with single cells in gel injection. Representative H&E images of single and progressive cells in gel injections were shown in upper row of (**c**). Scale bars, 1 mm. Implants were stained for GFP (green) to identify injected cells and their representative images were shown in lower row of (**c**). Implanted site of single cells in gel were distinguished by white dash line. Scale bars, 200 μm.

**Figure 3:**
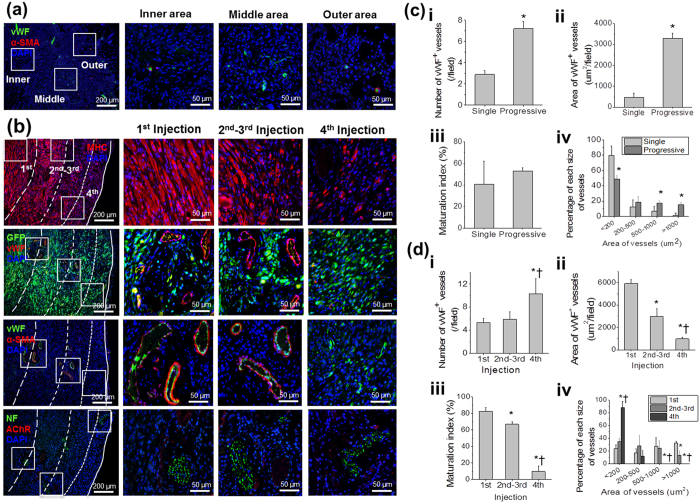
Volumetric muscle tissue construction and vascularization by multiple cell injections in a progressive manner. (**a**) Representative vWF (green)/α-SMA (red) staining images of implants constructed by single injection. (**b**) Representative staining images of implants constructed by 4-progressive injection. MHC (red) in the first row, GFP (green)/vWF (red) in the second row, vWF (green)/α-SMA (red) in the third row, NF (green)/AChR (red) in the fourth row. Low magnification images of implants were shown in left column. Scale bars, 200 μm. High magnification images of the firstly, secondary and thirdly, and fourthly injected areas were shown in second, third and fourth columns, respectively. Scale bars, 50 μm (x400 magnification). (**c,d**) *In-vivo* vascularization of single injection vs progressive injection (**c**) and each injected area in progressive injection (**d**) was evaluated with staining images for vWF/α-SMA (x400 magnification) in aspects of number of vWF^+^ vessels (per field, **i**), area of vWF^+^ vessels (μm^2^ per field, **ii**), maturation index (%, **iii**) and percentage of each size of vessels (**iv**). Maturation index (%) = α-SMA^+^ vessels/total vessels × 100. (**c**) Student’s *t*-test (*n* = 4, 3–4 fields of each sample), *P < 0.05 with single injection. (**d**) ANOVA, Tukey test (*n* = 4, 3–5 fields per each area in each sample). *P < 0.05 with the 1^st^ injection, ^†^*P* < 0.05 with the 2^nd^–3^rd^ injection.

Another parameter for successful cell injection strategy involves an accurately controlled injection volume to reduce cell necrosis after cell transplantation. Generally, the implanted cells can obtain an adequate oxygen and nutrient supply within 200 μm from adjacent vasculatures as well as through diffusion at a distance of 0.2–0.3 cm^1^,^34^. While cell density was optimized at 10 × 10^6^ cells per ml for injection (see Supplementary Fig. S1), the higher cell injection volume in the single injection resulted in significantly lower cell survival compared to progressive injection (Fig. 2c). Thus, injection volume can affect cell survival. Since the volume of 1.2 ml for the single injection can prepare 1.2 cm^3^ in dimension (>1 × 1 × 1 cm), the cells localized to the center of the fibrin gel will encounter a hypoxic condition with limited oxygen diffusion from the host vessels^1^,^15^,^16^,^20^, and the result will be increased cell necrosis. Eventually, the increased cell death will lead to the failure of the volumetric tissue construction (Fig. 2c, single injection). Meanwhile, the volume of each injection in the progressive injection model was 0.3 ml and is equivalent to 0.3 cm^3^, which can reconstruct an implant of approximately 300 mm^3^ in volume due to the diffusion of oxygen from the surrounding blood vessels. The injected cells obtain an adequate blood supply through diffusion and have greater survival. Therefore, the volume for injection should be controlled to ensure cell survival and subsequent formation of tissue reconstruction within the implant site (Fig. 3).

Optimization of several parameters such as cell density, time interval between cell injections, and volume for cell injections allowed us to demonstrate the possibility to use a progressive cell injection model to facilitate volumetric muscle tissue construction through efficient vascularization events in an ectopic site (Fig. 3). We show that the levels of vascularization in the progressive cell injection group were significantly higher than that of the single injection group in the ectopic implantation study. Notably, the vascular formation pattern in the core region was significantly different. While the progressive cell injections resulted in the formation of larger (>500 μm) and mature blood vessels in the core region, the area of blood vessels present in the single injection group was significantly lower by a 300-fold difference. Interestingly, the majority of the blood vessels (90%) found in the single cell injection group consisted of small size capillaries (<200 μm) (Fig. 3 and Supplementary Fig. S3). As such, it is speculated that the progressive cell injection strategy facilitates the formation of volumetric viable tissue by establishing vascular networks throughout the tissue construct, even in the core region, thus overcoming the problems resulting from the conventional single cell injection method, such as necrotic tissue core due to diffusion limitation.

**Figure 4:**
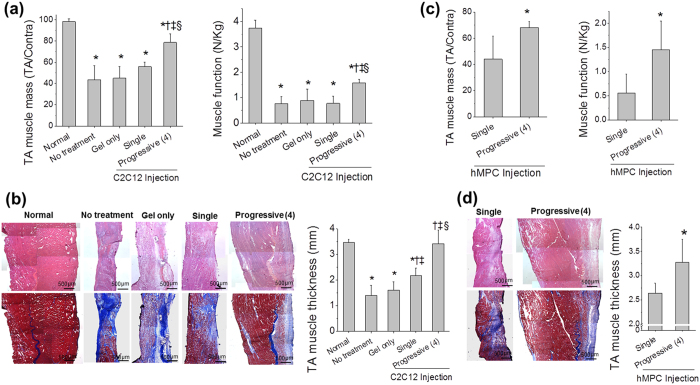
Volumetric skeletal muscle tissue reconstruction with recovery of muscle function by multiple cell injections in a progressive manner in the injury model of volumetric muscle loss. Tibialis anterior (TA) muscle injury was created in nude rats and 4-progressive injection of C2C12 (**a,b**) and hMPCs (**c,d**) were performed. The TA muscles were harvested at 1 week after the fourth injection. (**a,c**) TA muscle mass (defected TA per Contralateral TA) and muscle force (N Kg^−1^) of C2C12 injection in (**a**) and hMPC injection in (**c**). (**b,d**) Representative H&E and masson’s trichrome staining images and TA muscle thickness (mm) of C2C12 injection in (**b**) and hMPC injection in (**d**). The TA muscle thickness (mm) was measured by using H&E staining images. Three different injected areas of the TA per sample were chosen to measure the TA muscle thickness. H&E staining images in upper row. Masson’s trichrome staining images in lower row. Scale bars, 500 μm. (**a**) ANOVA, Tukey test (*n* = 4). In TA muscle mass, *P < 0.05 with Normal, ^†^*P* < 0.001 with No treatment, ^‡^*P* < 0.001 with Gel only, ^§^*P* < 0.017 with Single C2C12. In muscle function, *P < 0.001 with Normal, ^†^*P* < 0.015 with No treatment, ^‡^*P* < 0.045 with Gel only, ^§^*P* < 0.016 with Single C2C12. (**b**) ANOVA, Tukey test (*n* = 4). *P < 0.001 with Normal, ^†^*P* < 0.001 with No treatment, ^‡^*P* < 0.003 with Gel only, ^§^*P* < 0.001 with Single C2C12. (**c**) Student’s *t*-test (*n* = 4). *P < 0.045 with Single hMPC in TA muscle mass, *P < 0.038 with Single hMPC in muscle function. (**d**) Student’s *t*-test (*n* = 4). *P < 0.003 with Single hMPC.

**Figure 5:**
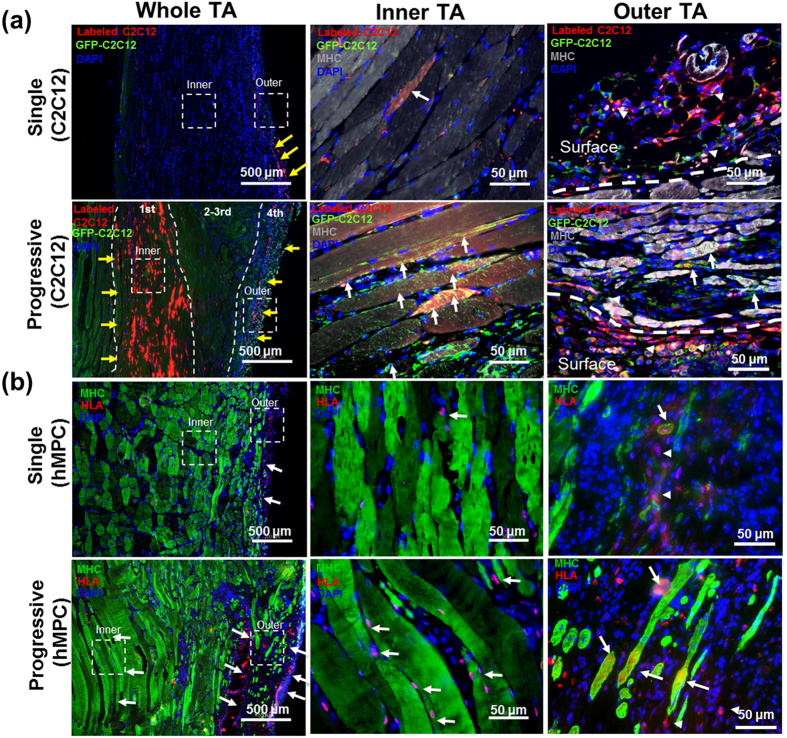
Identification of injected cells and their myotubes or myofibers formation in the injured muscles. (**a**) Representative staining images of single-C2C12 and progressive-C2C12 injected TA muscles. For both single and progressive injection, GFP^+^ -C2C12 was injected and identified by staining for GFP (green). Myotubes or myofibers were stained for MHC (grey). GFP^+^ -C2C12 was labeled with DiI (red) in single-C2C12 injection. In progressive-C2C12 injection, the firstly and fourthly injected GFP^+^ -C2C12 were labeled with DiI (red). Yellow arrows, GFP^+^/DiI^+^ cells. White arrows, MHC^+^/GFP^+^/DiI^+^ cells. White arrowheads, MHC^-^/GFP^+^/DiI^+^ cells. (**b**) Representative staining images of single-hMPC and progressive-hMPC injected TA muscles. Injected hMPC were identified by HLA staining (red) and myotubes or myofibers were stained for MHC (green). White arrows, MHC^+^/HLA^+^ cells. (**a,b**) Scale bars, 500 μm in left column and 50 μm in middle and right columns.

**Figure 6:**
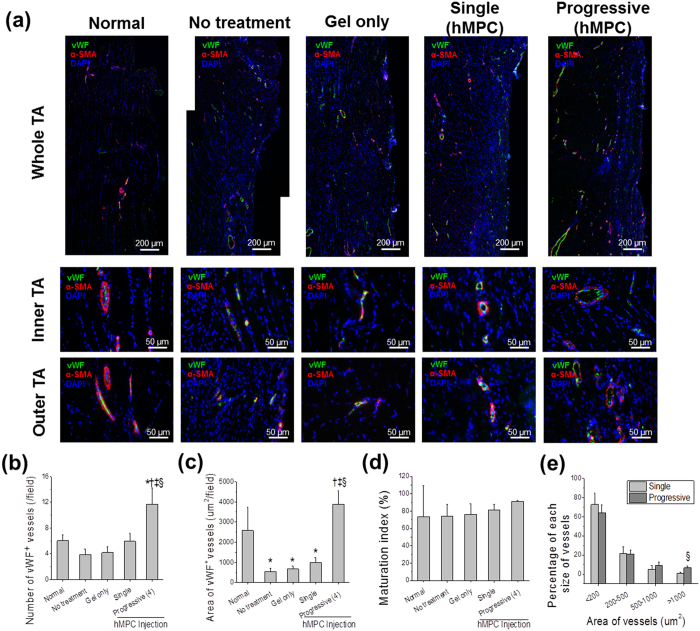
Vascularization by multiple cell injection in a progressive manner in the injury model of volumetric muscle loss. (**a**) Representative vWF (green)/α-SMA (red) staining images of hMPCs-injected TA muscles. Low magnification images of implants were shown in upper row. Scale bars, 200 μm. High magnification images of inner and outer TA muscles were shown in middle and lower rows, respectively. Scale bars, 50 μm (x400 magnification). (**b**–**e**) Vascularization of each group was quantified with staining images for vWF/α-SMA (x400 magnification) in terms of number of vWF^+^ vessels (per field, **b**), area of vWF^+^ vessels (μm^2^ per field, **c**), maturation index (%, **d**) and percentage of each size of vessels (**e**). Maturation index (%) = α-SMA^+^ vessels/total vessels × 100. ANOVA, Tukey test (*n* = 4, 6–10 fields of each sample). *P < 0.018 with Normal, ^†^*P* < 0.001 with No treatment, ^‡^*P* < 0.001 with Gel only, ^§^*P* < 0.001 with Single hMPCs.

Encouraged by the promising outcomes, this novel cell delivery system was applied to treat a critical-sized muscle tissue defect. As the first target for reconstruction of damaged tissues or organs, the strategy of progressive cell injections was applied to skeletal muscle tissue injuries, particularly VML, which is caused by traumatic or surgical loss. VML is a challenging clinical problem for military, civilian, and sports medicine since skeletal muscle is a relatively large, thick tissue that often involves other tissue or organ damage such as skin, bone, and internal organs^35^. As an animal model for VML, we utilized a rat TA muscle defect model that was developed by Wu, X. *et al*^36^. after modification. This is a standardized rodent model of VML injury generated by excising ~20% of the middle of the TA muscle, and muscle weight and tetanic muscle force are not recovered until 6 months^36^,^37^. In this study, we introduced a larger defect size (~30% excision of TA muscles) to produce a critical sized muscle defect animal model. The anatomical and functional analysis demonstrated that our progressive cell injection model could significantly increase muscle mass and thickness, reduce fibrosis and partial restoration of muscle function in the TA defect when compared to that of a single injection (Fig. 4); moreover, recovery was confirmed by myotubes or myofibers formation by progressively injected cells (Fig. 5 and Supplementary Figs S6 and S7) as well as vascularization (Fig. 6) and neuronal ingrowth (see Supplementary Fig. S8).

Multiple cell injections in a progressive manner showed better cell engraftment over the single injection method, as evidenced by the presence of numerous GFP^+^ or DiI^+^ cells and HLA^+^ or HNA^+^ cells in the progressive cell injection group, as compared with those in the single cell injection group. In addition, higher proliferation of the engrafted cells (30% of engrafted hMPCs) was observed in the progressive injection group than in the single cell injection group. Based on these observations, it is speculated that if the same number of cells are injected, the strategy of progressive cell injections would increase cell engraftment and proliferation, as compared to the single injection method. Moreover, the myogenic capacity of engrafted cells would be increased by the progressive injection strategy. The percentage of differentiating cells in the progressive-C2C12 and hMPCs injection was 43.74% and 19.18%, respectively, at 1 week after the final injection.

While the current study showed the significant potential of the progressive cell injection strategy, several limitations remain to be solved before the application could be translated. Since volumetric tissue repair and functional recovery was evaluated only up to 4 weeks in a VML injury model, reliability of the therapeutic effects need to be demonstrated over a long-term. In terms of defect size, in this study, we used a TA injury model of approximately 30% muscle mass defect. Although the extent of defect is significant in a rodent model, it is unclear whether this defect size reflects the clinical conditions presented in humans. Further investigations using a larger animal model with critical defect size and mass^38^ should be performed to determine the effectiveness of the progressive cell injections strategy. Muscle function is closely related to innervation and anti-fibrosis; therefore, specific factors that facilitate efficient innervation and reduction in fibrosis should be considered to improve muscle function. Such factors include agrin^39^ or suramin^40^ to accelerate innervation or reduce fibrosis, respectively.

This manuscript describes a proof-of-concept study showing that multiple cell injections result in enhanced cell survival than single injection, which contributes to improved muscle function structurally and physically. To prove the hypothesis, we developed a multiple injection protocol that is performed in a progressive manner with several cell injection parameters. Although we have obtained positive outcomes, in terms of muscle recovery using a pre-clinical animal model, translation of this technology into the clinical settings requires further optimization and refinement, as well as validation in a clinically relevant animal model. For example, translation of this technique to clinical applications needs modification of the cell injection parameters, depending on the target tissue and defect size including injection volume, injection time interval and cell concentration. Selection of a cell delivery vehicle should also be considered for clinical translation. In this study, fibrin gel was used as a cell delivery vehicle since it has been widely used in various clinical applications. Since a hydrogel system such as fibrin gel usually displays weakness in mechanical property, other biocompatible materials with enhanced mechanical strength should be identified in order to maintain the implant volume for a longer period of time.

In conclusion, our study provides a novel cell delivery strategy utilizing an appropriate and efficient *in vivo* vascularization process to overcome the reduced cell survival limitation of current cell-based therapies. The concept of “multiple and progressive cell injections” was supported by demonstrating that the progressive cell injections resulted in an improved cell survival through normal and efficient angiogeneic events surrounding the implant and led to reconstruction of volumetric muscle tissues *in vivo*. In addition, this novel strategy was applied to a critical-size muscle defect to show restoration of muscle mass and function in a VML animal model. Therefore, multiple cell injections in a progress manner present a promising strategy for volumetric tissue repair in TE and RM.

## Methods

### Cell culture and materials preparation

C2C12 mouse myoblasts (ATCC, Manassas, VA) were transduced with GFP to prepare GFP^+^ -C2C12 with a method developed previously^41^. GFP^+^ -C2C12 cells were cultured in DMEM/high glucose (Thermo Scientific Inc., Waltham, MA) supplemented with 10% fetal bovine serum (FBS, Gibco, Carlsbad, CA) and 1% penicillin/streptomycin (PS, Thermo Scientific) at 37 °C with 5% CO_2_. During the cell culture, the GFP expression of C2C12 cells was confirmed by a fluorescent imaging. As another cell source for this study, hMPCs were used after isolation and expansion. hMPC were isolated from human muscle biopsies as previously described^42^ and expanded in a growth medium composed of DMEM/high glucose, 20% FBS, 2% chicken embryo extract (Gemini Bio-Products, West Sacramento, CA) and 1% PS. Cells were expanded up to passage 4 for cell injection study. As a vehicle for the cell injection, a fibrin gel system was used. To form the fibrin gel, 40 mg ml^−1^ of fibrinogen solution and 40 U ml^−1^ of thrombin solution (Sigma, St. Louis, MO) were prepared by dissolving fibrinogen from bovine plasma (Sigma) in 0.9% sodium chloride saline solution, and bovine thrombin (Sigma) in 25 mM CaCl_2_ in saline solution, respectively. For cell injection, the muscle cells were suspended with fibrinogen solution and adjusted to a cell concentration of 20 × 10^6^ cells per ml to yield a final cell concentration of 10 × 10^6^ cells per ml in the fibrin gel after mixing with thrombin solution at a 1:1 ratio.

### Ectopic cell injection

All animal procedures were performed in accordance with a protocol approved by the Institutional Animal Care and Use Committee at Wake Forest University School of Medicine. Male athymic mice (6–8 weeks old, total 24 mice) were obtained from Charles River Laboratory (Wilmington, MA). Anesthesia was induced by using 3% isoflurane before surgical procedures. Under aseptic conditions, subcutaneous injections into the dorsal to dorso-lateral region were performed. For progressive cell injections, C2C12 cells in a fibrin gel were delivered into the left dorsal regions of mice; 2, 4, 6, and 8 injections (*n* = 3–4). Injections were performed every 7 days at the same site where the former injection was done. As control, the same volume of gel without cells was injected into the contralateral region of each animal in the same manner. For each injection, 150 μl volume of fibrinogen solution with or without cells was injected using a 26-gauge needle. An equal volume of thrombin solution was immediately injected at the same site, where the injected cells will be placed within the fibrin gel. Animals were euthanized 1 week after the final injection. To determine efficiency of progressive cell injection, the volume of implant after 4 injections was evaluated and the measured volume was compared with that by single injection of cells. For the single injection, 600 μl of fibrinogen solution with cells and 600 μl of thrombin solution were injected, of which number of cells is equal to the total number of 4 injections. The single or progressive injection group was euthanized 4 weeks after injection or 1 week after the 4^th^ injection, respectively. Implant volume was measured by water displacement method, then implant was evaluated by histological and immunohistological analysis^43^.

### VML injury model and cells injection

The VML injury model was created in nude rats (male, 12–14 weeks old, Charles River Laboratory)^36^. Under anesthesia, the fascia was separated from the TA muscle, and then approximately 30% of middle third TA muscle was excised. The excised TA muscle weight was estimated by following using the equation: y (g) = 0.0017 × body weight (g) – 0.0716. In addition to the TA muscle excision, extensor digitorum longus (EDL) and extensor hallucis longus (EHL) muscles were removed to exclude compensatory hypertrophy during muscle regeneration following TA excision. The remaining TA muscle was covered with fascia and skin was closed using sutures and surgical glue. Fibrin gels with or without cells were injected into the defect sites. In this study, 7 groups were investigated (*n* = 4 per group, total 28 rats); (1) normal (age-matched control), (2) no treatment (defect only), (3) multiple injection-gel only, (4) single injection-C2C12, (5), progressive injection-C2C12, (6) single injection-hMPC, (7) progressive injection-hMPC. For a single injection, 300 μl of fibrinogen solution with cells was delivered into the defect sites with a 26-gauge needle followed immediately by an additional injection of 300 μl of thrombin solution at the same injection site to form fibrin gel (total injection volume = 600 μl). The volume of 600 μl filled the defect in the TA muscle. For multiple and progressive injections, 4 cell injections were performed every week. The first injection was performed with a total volume of 300 μl and subsequent three injections were done with a volume of 100 μl per injection. To track the injected cells within the TA muscle, C2C12 cells were labeled with DiI (Vybrant^®^ Multicolor Cell-Labeling Kit, Thermo Scientific, Inc.) for the 1^st^ and 4^th^ injections and co-labeling of DiI and GFP was used to identify the injected C2C12 cells within the TA defect.

### *In vivo* functional analysis of TA muscle

To examine restoration of muscle function, tetanic force of TA muscle was measured at 4 weeks after surgery (1 week after 4^th^ injection in the multiple and progressive injection). Anterior crural muscle *in vivo* mechanical properties were analyzed with the dual-mode muscle lever system (Aurora Scientific, Inc., Mod, 305b, Aurora, Canada)^36^. The foot to be measured was attached to a foot plate and knee and ankle were positioned at 90-degree angle. Tetanic analysis was performed by stimulating the peroneal nerve using a Grass stimulator (S88) at 100 Hz with a pulse-width of 0.1 msec and 10 V. Muscle force (N Kg^−1^) was calculated by peak isometric torque per body weight (*n* = 4 per group). After the functional assessment and harvesting of TA muscle, the retrieved TA muscle tissue was weighed and processed for histological analysis. The percentage of muscle mass was calculated by the ratio of the weight of injured TA muscle to that of contralateral TA muscle (*n* = 4 per group).

### Histological and immunofluorescent analysis

The harvested TA muscles were freshly frozen in liquid nitrogen immediately for cryo-embedding or fixed with 4% paraformaldehyde for paraffin embedding. For histological evaluations, H&E staining and MT staining were performed on the tissue sections. To evaluate TA muscle thickness of each group, three different regions in the middle of the TA muscles in the H&E images were chosen and the thickness was measured (*n* = 4 of each per group) in blinded fashion.

For immunostaining, the cryosections (7 μm) were fixed with 4% paraformaldehyde. Paraffin sections (5 μm) were deparaffinized and processed for antigen retrieval with the heat-induced process using sodium citrate buffer. Tissue sections were incubated with methanol at −20 °C for 10 minutes, acetone at room temperature for 7 minutes or 0.2% Triton X-100 for 30 minutes at room temperature for permeabilization, and then blocked using a serum-free blocking agent (X090930-1; Dako, Carpentaria, CA) for 1 h at room temperature. All antibodies were diluted with antibody diluent (S302283-1; Dako), and the blocked sections were incubated with primary antibodies at room temperature for 1 h or incubated at 4 °C for overnight. Secondary antibodies such as Alexa 488-conjugated anti-mouse or anti-rabbit antibody (A11017; A11070; 1:200 dilution; Invitrogen, Eugene, OR), Texas Red-conjugated anti-mouse, anti-rabbit, or anti-rat antibody (TI-2000; TI-1000; TI-9400; 1:200 dilution; Vector Labs, Burlingame, CA), or Cy5-conjugated anti-mouse or anti-rabbit antibody (A10524; A10523; 1:200 dilution; Invitrogen) were treated at room temperature for 40 min. Tissue sections were then mounted with VECTASHIELD Mounting Media with DAPI (H-1200; Vector Labs) and analyzed by fluorescent imaging using an upright (LEICA) and confocal microscope (Olympus).

To identify the injected GFP^+^ -C2C12 cells, the sections were incubated with mouse anti-GFP antibody (sc-9996; 1:100 dilution; Santa Cruz Biotechnology, Santa Cruz, CA) or rabbit anti-GFP antibody (ab290; 1:500 dilution; Abcam, Cambridge, MA). For hMPC tracking, tissues were stained with rabbit anti-HLA A (ab52922; 1:100 dilution; Abcam) or mouse anti-HNA (MAB1281; 1:100 dilution; EMD Millipore, Darmstadt, Germany). Muscle differentiation was examined by immunostaining for mouse anti-MHC (MF20; 1:30 dilution; Developmental Studies Hybridoma Bank, Iowa City, IA) or mouse or rabbit anti-Myogenin (ab1835; 1:100 dilution, ab124800; 1:200 Abcam). Cells proliferation was estimated by immunostaining for mouse or rabbit anti-PCNA (ab29; 1:500 dilution, ab18197; 1:1000 dilution, Abcam). To evaluate muscle fibrosis, tissue sections were stained with rabbit anti-collagen I (ab34710; 1:200 dilution; Abcam). For vascular integration, the tissue sections were stained with rabbit anti-vWF (A0082; 1:400 dilution; DAKO) and mouse anti-α-SMA (sc-32251; 1:50 dilution; Santa Cruz). Neuronal ingrowth was visualized by immunostaining with rabbit anti-NF (N4142; NF200, 1:80 dilution; Sigma) and rat anti-AChR (ab24719; 1:100 dilution; Abcam).

To evaluate vascularization and the vascular maturation, vWF/α-SMA double immunostaining was performed and using 3 to 5 randomly selected images from each injected regions of the retrieved tissues (x400 magnification, *n* = 4 per group)^44^. The number of vWF^+^ vessels (per field of 400x magnified image), area of vWF^+^ vessels (μm^2^ per field of 400x magnified image), and percentage of each size of vessels were quantified and the maturation index (%) was determined as the ratio of α-SMA^+^ vessels to the total number of vessels^45^,^46^. Blood vessels were quantitatively analyzed with image analysis software (Image J) in blinded fashion. To identify whether the infused cells contributed to formation of muscle fibers *in vivo*, GFP/MHC and HLA/MHC staining was performed on the GFP^+^ -C2C12- and hMPC-injected TA muscles, respectively. Double positive staining in the fluorescent images was considered to be a contribution of the injected cells in the muscle fiber formation. Myogenesis of injected cells were also identified by Myogenin^+^/DiI-labeled cells in C2C12-injected TA muscles and Myogenin^+^/HNA^+^ cells in hMPC-injected TA muscles (x400 magnification, *n* = 3–4 per group, 3–5 fields per each sample). Proliferation of injected C2C12 and hMPCs were evaluated by double positive of PCNA/DiI-labeled cells in C2C12-injected TA muscles and PCNA/HNA cells in hMPC-injected TA muscles (x400 magnification, *n* = 3–4 per group, 3–5 fields per each sample). TA muscle fibrosis of inner and outer area was quantified by percentage of collagen I positive area in the fluorescent images of collagen I/MHC staining (x200 magnification, *n* = 4 per group, 3 fields of each area per each sample).

### Statistical analysis

Results were analyzed with Origin Pro 8.5 (OriginLab Co, Northampton, MA, USA) and SPSS software (SPSS, version 19; IBM, Armonk, NY). One-way analysis of variance (ANOVA), Tukey *post hoc* testing and Student’s t-test were applied to mean comparisons. Variables are expressed as mean ± standard deviation, and differences were considered significant at *P* < 0.05.

## Additional Information

**How to cite this article**: Kim, J. H. *et al*. Progressive Muscle Cell Delivery as a Solution for Volumetric Muscle Defect Repair. *Sci. Rep.* **6**, 38754; doi: 10.1038/srep38754 (2016).

**Publisher's note:** Springer Nature remains neutral with regard to jurisdictional claims in published maps and institutional affiliations.

## Supplementary Material

Supplementary Information
