# Supplementary material for: Progressive Muscle Cell Delivery as a Solution for Volumetric Muscle Defect Repair
Source: Sci Rep. 2016 Dec 7;6:38754. doi: 10.1038/srep38754 (PMC5141432; doi:10.1038/srep38754)
Supplement: Supplementary Information [file srep38754-s1.pdf]

Progressive Muscle Cell Delivery as a Solution for Volumetric Muscle Defect Repair

Ji Hyun Kim[1,†], In Kap Ko[1,†], Anthony Atala[1] & James J. Yoo[1,*]

[1] Wake Forest Institute for Regenerative Medicine, Wake Forest School of Medicine, Winston-Salem, NC 27157, USA

[†]These authors contributed equally to this work.

*Correspondence and requests for materials should be addressed to J.J.Y.

James J. Yoo, M.D., Ph.D.

Wake Forest Institute for Regenerative Medicine

Wake Forest School of Medicine

Medical Center Boulevard

Winston-Salem, North Carolina 27157

USA

Tel: +1-336-713-7294/Fax : +1-336-713-7290

E-mail: jyoo@wakehealth.edu

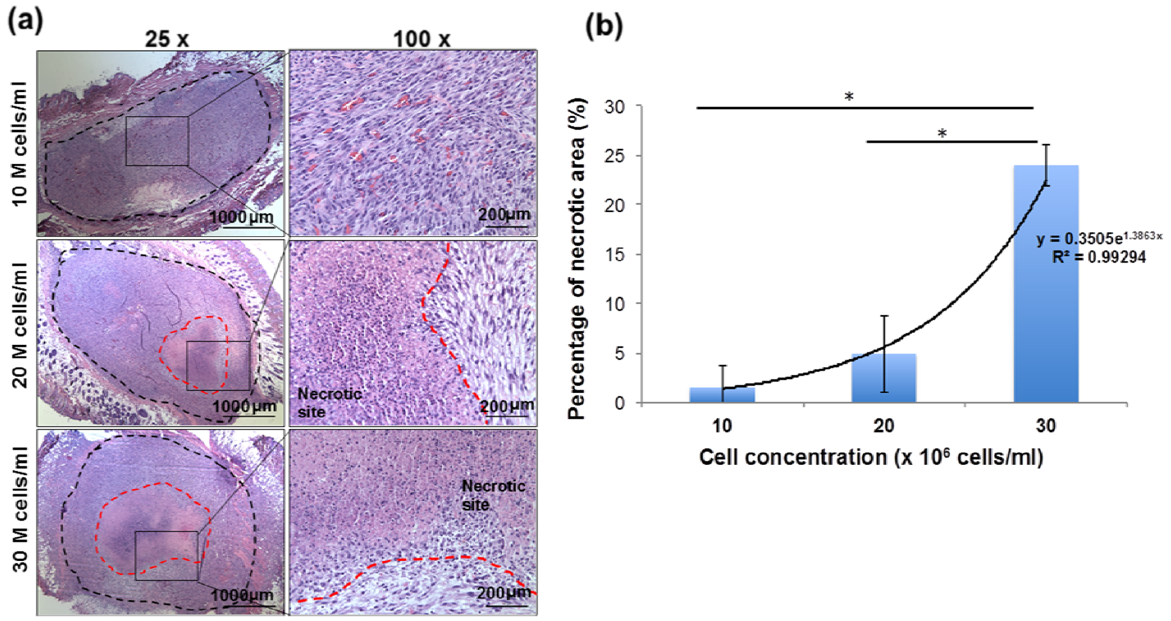

Supplementary Figure S1. Limitation of cell survival following transplantation. C2C12 in fibrin gel which had different cell concentration, 10, 20, and 30×10^6 cells per ml, were subcutaneously implanted into athymic mice, and necrotic areas of implants were measured by H&E staining images. (**a**) Representative H&E staining images of implants. Implanted site was marked by black dash line and necrotic site was distinguished by red dash line. Scale bars, 1000 μm in left column and 200 μm in right column. (**b**) Percentage of necrotic area (%). ANOVA, Tukey test ($n = 3$). *P<0.05.

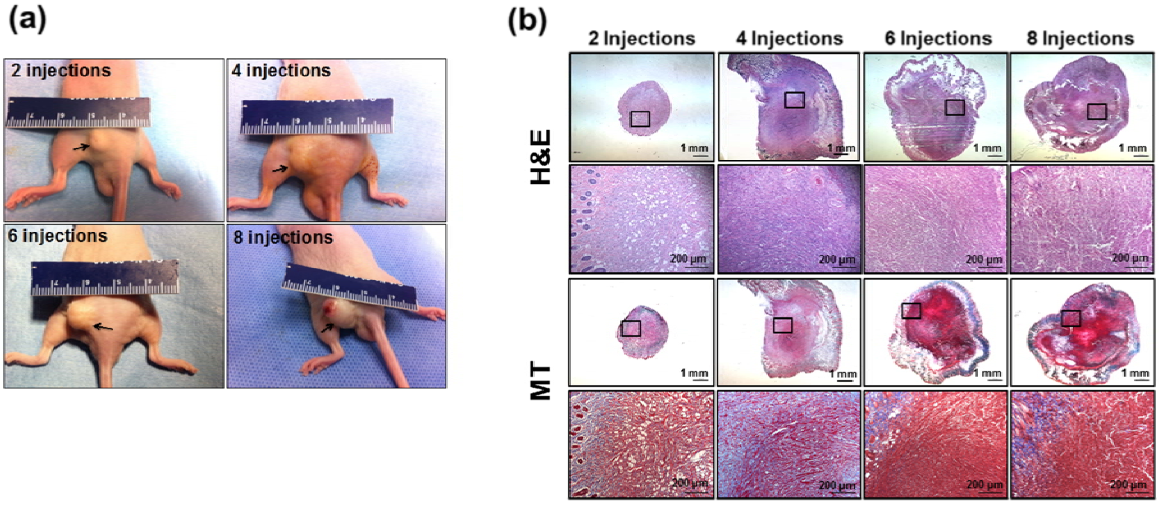

Supplementary Figure S2. Ectopic muscle construction by multiple cell injections in a progressive manner. C2C12 in gel was subcutaneously injected into athymic mice (progressive 2, 4, 6 and 8 injections) and the ability of the volumetric muscle tissue construction was evaluated histologically. (**a**) Representative pictures of implants. Black arrows, implants. (**b**) Representative images of H&E and masson's trichrome staining. Scale bars, 1 mm in the first and third rows and 200 µm in the second and fourth rows.

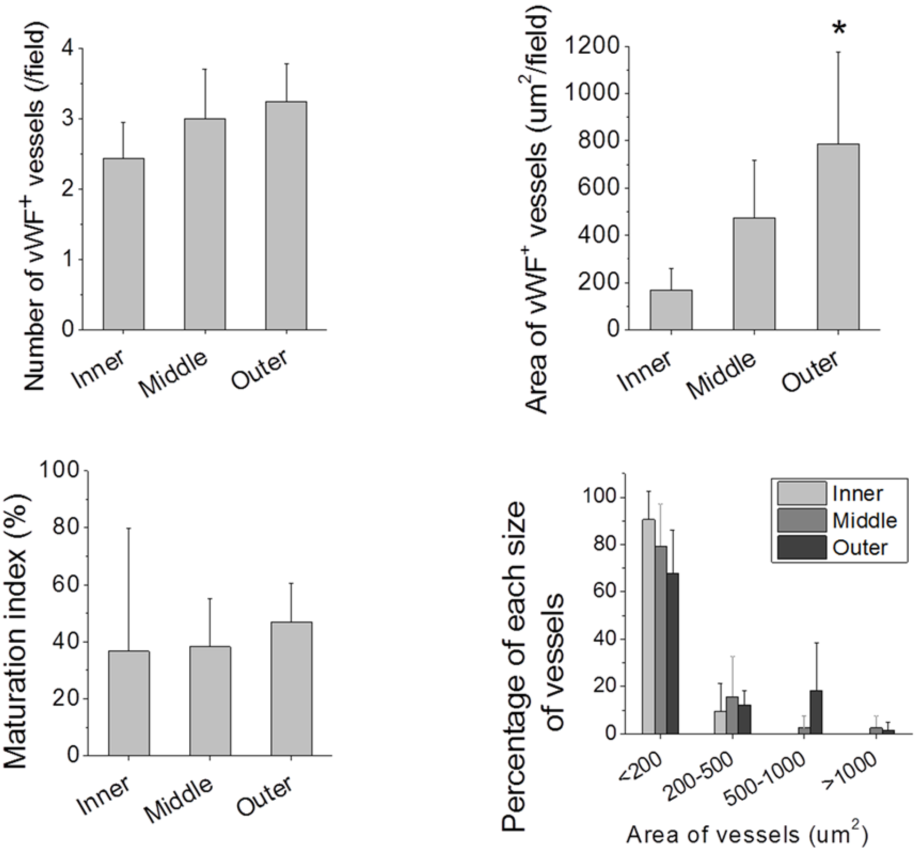

Supplementary Figure S3. Vascularization by single cell injection. C2C12 cells were injected in ectopic site and the vascularization of inner, middle and outer areas of the implants were evaluated with staining images for vWF/α-SMA (x400 magnification) in terms of number of vWF^+ vessels (/field), area of vWF^+ vessels (μm^2/ field), maturation index (α-SMA^+ vessels / total vessels × 100, %) and percentage of each size of vessels. ANOVA, Tukey test ($n = 4$, 3-4 fields per each sample and each area). *$P<0.025$ with inner area.

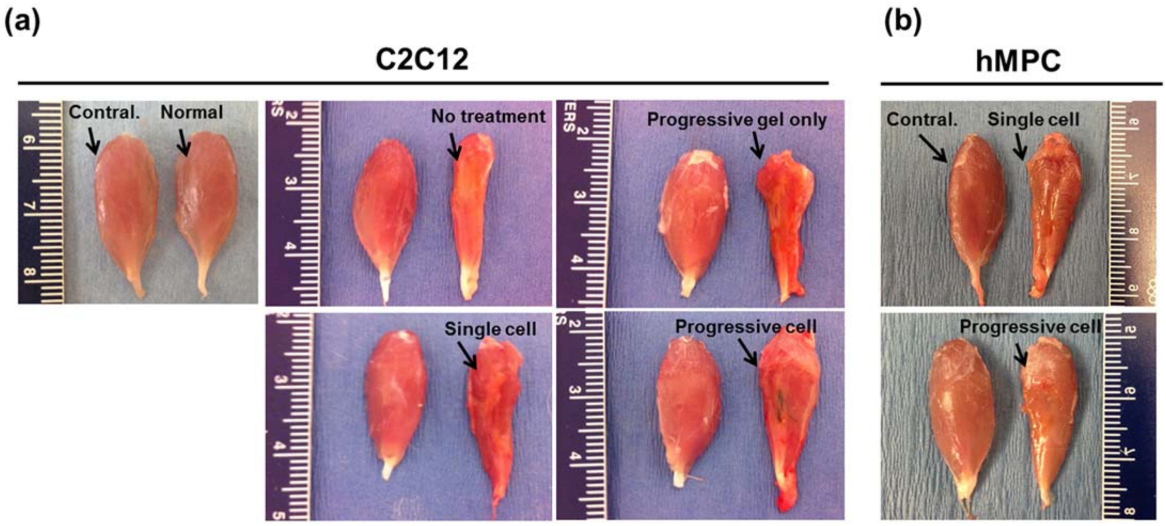

Supplementary Figure S4. Gross images of harvested TA muscles. To create injury model of volumetric muscle loss, 30% of TA muscle of left leg in nude rat was excised. For single cell injection, cells in gel were injected into the defect site following TA muscle removal. For progressive injection, cells in gel or gel only were injected every 1 week. TA muscles of left leg (treated TA) and right leg (contralateral TA) were harvested at 1 week after the fourth injection in progressive injection group and harvested at 4 weeks after surgery in no treatment group and single cell injection group. (**a**) C2C12-injected TA muscles. (**b**) hMPC-injected TA muscles.

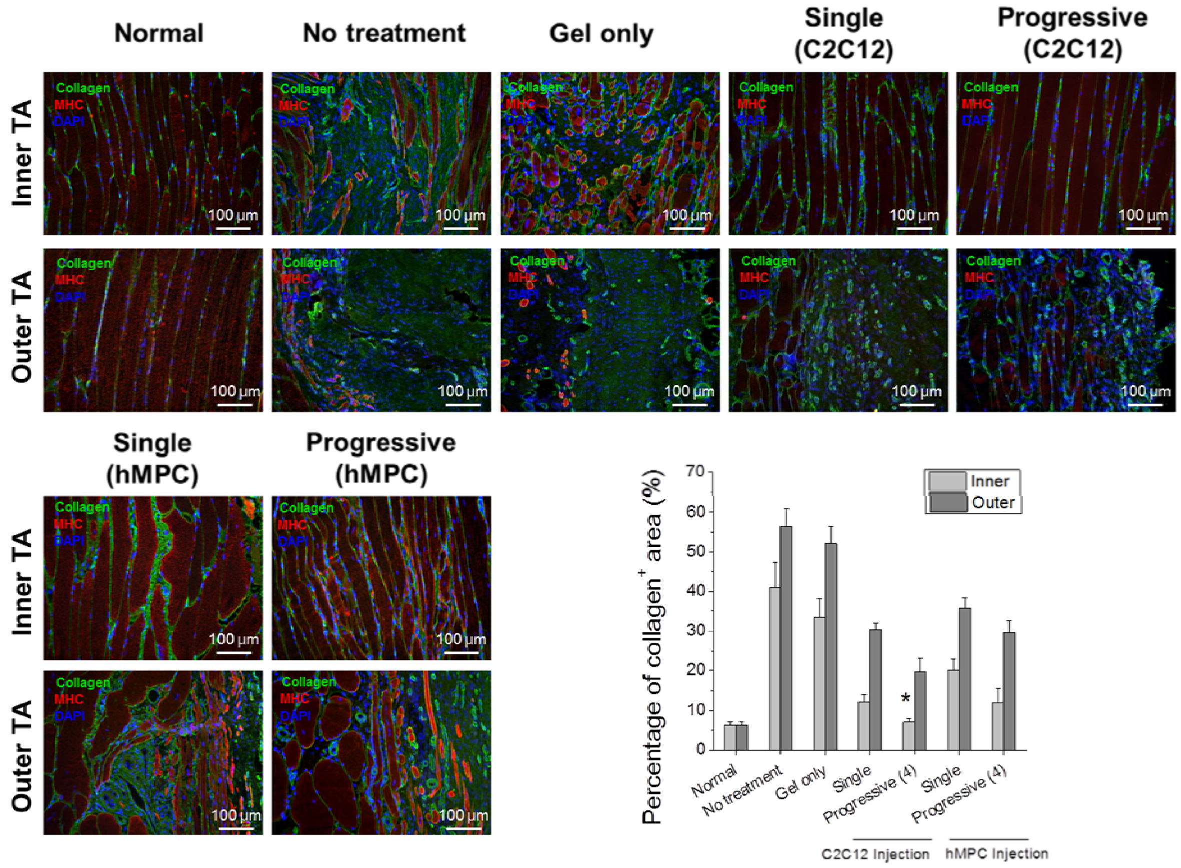

Supplementary Figure S5. Fibrosis of reconstructed TA muscles by multiple cell injections in a progressive manner. Representative staining images with collagen (green)/MHC (red) of harvested TA muscles (x200 magnification). TA muscle fibrosis of each group was evaluated by percentage of collagen^{+} area (%). ANOVA, Tukey test (n = 4, 3 fields per each sample and each area). *P>0.997 with Normal. Scale bars, 100 µm.

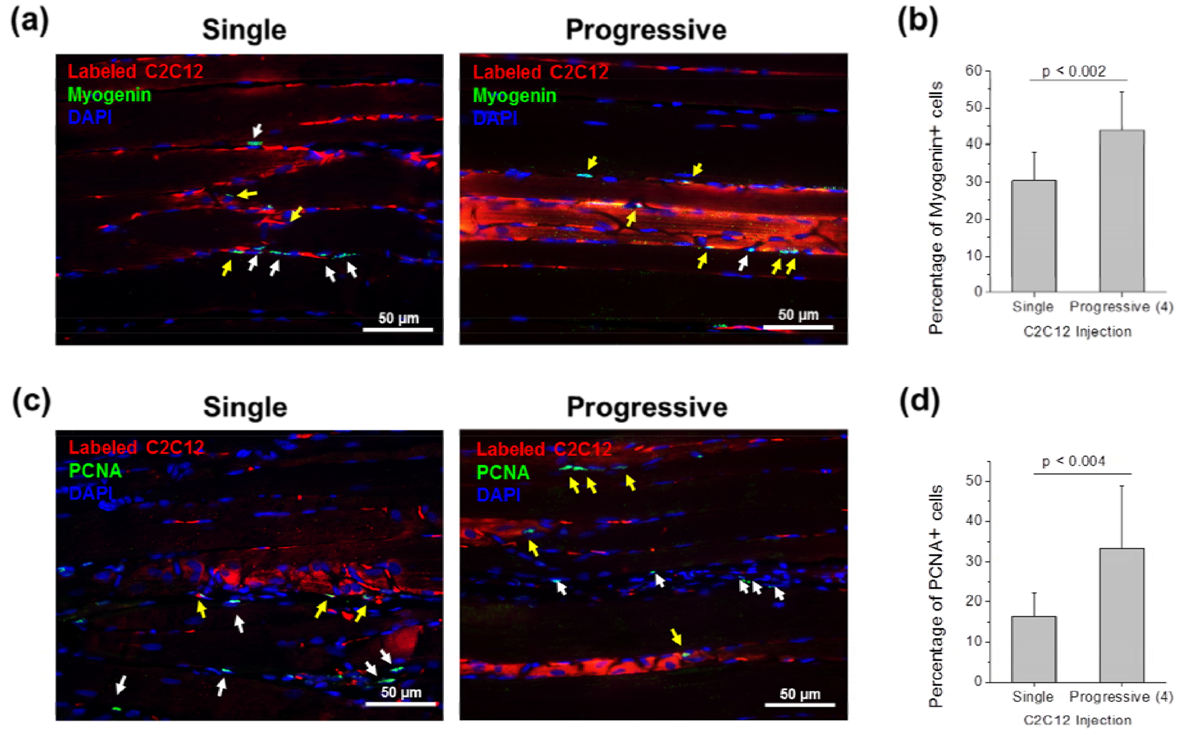

Supplementary Figure S6. Myotubes or myofibers formation and proliferation of C2C12 cells injected in the injured muscles. (**a** and **b**) Representative staining images with Myogenin (green, **a**) and PCNA (green, **c**) of single-C2C12 and progressive-C2C12 injected TA muscles. Injected C2C12 cells were labeled with DiI (red). (**b** and **d**) Percentage of differentiation (**b**) and proliferation (**d**) of injected cells. (**a** and **b**) Myotubes or myofibers formation of injected C2C12 cells were evaluated by Myogenin^{+}/DiI^{+} cells. Yellow arrows, Myogenin^{+}/DiI^{+} cells. White arrows, Myogenin^{+}/DiI^{-} cells. (**c** and **d**) Proliferating C2C12 cells were identified by PCNA^{+}/DiI^{+} cells. Yellow arrows, PCNA^{+}/DiI^{+} cells. White arrows, PCNA^{+}/DiI^{-} cells. Student's *t*-test (n = 3-4, 3-5 fields per each sample). Scale bars, 50 μm.

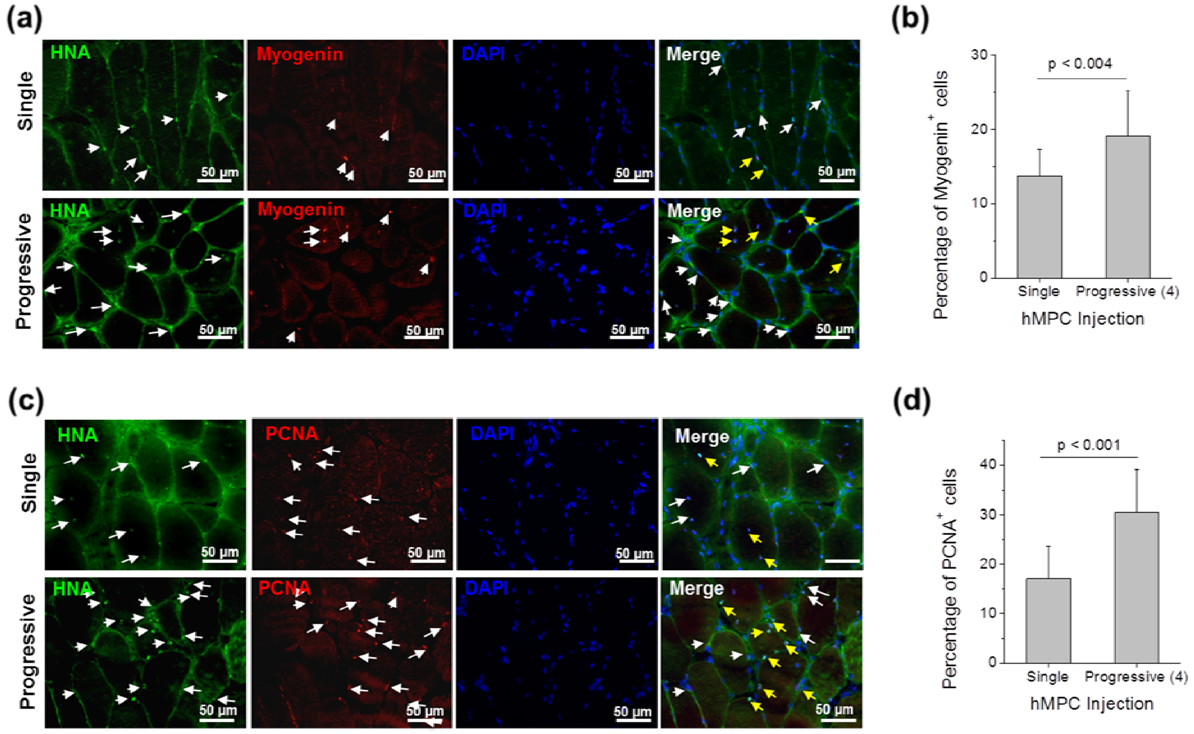

Supplementary Figure S7. Myotubes or Myofibers formation and proliferation of hMPCs cells injected in the injured muscles. (**a** and **c**) Representative staining images with Myogenin (red)/HNA (green) (**a**) and PCNA (red)/HNA (green) (**c**) of single-C2C12 and progressive-hMPCs injected TA muscles. Injected hMPCs were identified by staining for HNA (green). (**b** and **d**) Percentage of differentiation (**b**) and proliferation (**d**) of injected hMPCs. (**a** and **b**) Myotubes or myofibers formation of injected hMPCs were evaluated by $Myogenin^{+}/HNA^{+}$ cells. Yellow arrows, $Myogenin^{+}/HNA^{+}$ cells. White arrows, $Myogenin^{+}/HNA^{-}$ cells. (**c** and **d**) Proliferating C2C12 cells were identified by $PCNA^{+}/HNA^{+}$ cells. Yellow arrows, $PCNA^{+}/HNA^{+}$ cells. White arrows, $PCNA^{+}/HNA^{-}$ cells. Student's *t*-test (n = 3-4, 3-5 fields per each sample). Scale bars, 50 μm.

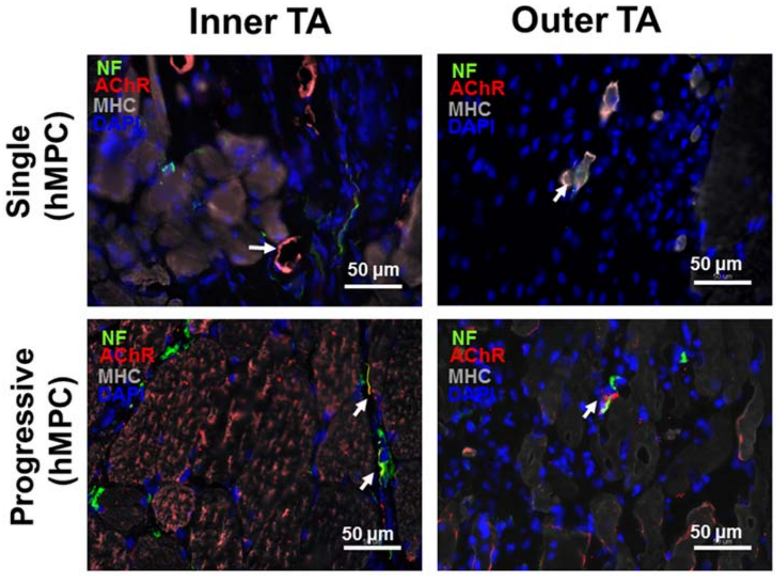

Supplementary Figure S8. Neuronal ingrowth of reconstructed TA muscles by single hMPC injection and multiple hMPC injections in a progressive manner. Representative staining images with NF (green)/AChR (red)/MHC (grey) of harvested TA muscles. White arrows, $NF^+/AChR^+/MHC^+$ cells. Scale bars, 50 µm.
